# Impact of 12-SNP and 6-SNP Polygenic Scores on Predisposition to High LDL-Cholesterol Levels in Patients with Familial Hypercholesterolemia

**DOI:** 10.3390/genes15040462

**Published:** 2024-04-06

**Authors:** Giovanna Cardiero, Martina Ferrandino, Ilenia Lorenza Calcaterra, Gabriella Iannuzzo, Matteo Nicola Dario Di Minno, Raffaele Buganza, Ornella Guardamagna, Renata Auricchio, Maria Donata Di Taranto, Giuliana Fortunato

**Affiliations:** 1 Dipartimento di Medicina Molecolare e Biotecnologie Mediche, Università degli Studi di Napoli Federico II, 80131 Naples, Italy; cardiero@ceinge.unina.it (G.C.); ferrandino@ceinge.unina.it (M.F.); giuliana.fortunato@unina.it (G.F.); 2 CEINGE-Biotecnologie Avanzate Franco Salvatore, 80145 Naples, Italy; 3 Dipartimento di Medicina Clinica e Chirurgia, Università degli Studi di Napoli Federico II, 80131 Naples, Italy; ilenialorenza.calcaterra@unina.it (I.L.C.); gabriella.iannuzzo@unina.it (G.I.); matteo.diminno@unina.it (M.N.D.D.M.); 4 Dipartimento di Scienze della Sanità Pubblica e Pediatriche, Università di Torino, 10126 Turin, Italy; raffaele.buganza@unito.it (R.B.); ornella.guardamagna@unito.it (O.G.); 5 Dipartimento di Scienze Mediche Traslazionali, Università degli Studi di Napoli Federico II, 80131 Naples, Italy; renata.auricchio@unina.it

**Keywords:** hypercholesterolemia, polygenic score, LDL-cholesterol, Familial hypercholesterolemia, phenotype variability

## Abstract

Background: Familial hypercholesterolemia (FH) comprises high LDL-cholesterol (LDL-c) levels and high cardiovascular disease risk. In the absence of pathogenic variants in causative genes, a polygenic basis was hypothesized. Methods: In a population of 418 patients (excluding homozygotes) with clinical suspicion of FH, the FH-causative genes and the regions of single nucleotide polymorphisms (SNPs) included in 12-SNP and 6-SNP scores were sequenced by next-generation sequencing, allowing for the detection of pathogenic variants (V+) in 220 patients. To make a comparison, only patients without uncertain significance variants (V−/USV−) were considered (*n* = 162). Results: Higher values of both scores were observed in V+ than in V−. Considering a cut-off leading to 80% of V−/USV− as score-positive, a lower prevalence of patients positive for both 12-SNP and 6-SNP scores was observed in V+ (*p* = 0.010 and 0.033, respectively). Mainly for the 12-SNP score, among V+ patients, higher LDL-c levels were observed in score-positive (223 mg/dL -IQR 187-279) than in negative patients (212 mg/dL -IQR 162–240; *p* = 0.006). Multivariate analysis confirmed the association of scores and LDL-c levels independently of age, sex, and presence of pathogenic variants and revealed a greater association in children. Conclusions: The 12-SNP and 6-SNP polygenic scores could explain hypercholesterolemia in patients without pathogenic variants as well as the variability of LDL-c levels among patients with FH-causative variants.

## 1. Introduction

Familial hypercholesterolemia (FH) is a genetic disease comprising high LDL-cholesterol (LDL-c) levels caused by pathogenic variants in genes involved in LDL uptake [1]. The heterozygous form of FH is a frequent disease with an estimated prevalence of about 1:250 subjects [2], whereas the homozygous form is very rare, with different reported prevalences in different populations [3,4,5]. The gene with the most pathogenic variants is the *LDLR* gene, which encodes the LDL receptor, whereas less frequent variants have been identified in genes encoding apolipoprotein B (*APOB*), i.e., the LDLR ligand and Proprotein Convertase Subtilisin/Kexin type 9 (*PCSK9*) [6,7]. The *LDLRAP1* gene is involved in the rare recessive form of FH [6,7]. Variants in other genes causative of different recessive disorders characterized by hypercholesterolemia have also been described in patients with clinical suspicion of FH; these genes were defined FH-phenocopies [8]. In particular, patients with heterozygous pathogenic variants in both FH-causative genes and genes causative of sitosterolemia (*ABCG5* and *ABCG8*) have been described as having a more severe phenotype than those with simple heterozygous FH [8]. This condition was defined as oligogenic FH and may be responsible for some of the phenotypic variability in FH [9].

High variability in LDL-c levels was observed among patients with genetically determined FH, only partially due to the variant type and the affected gene. In fact, patients with null variants in *LDLR* show higher LDL-c levels and an increased risk of cardiovascular diseases (CVDs) compared to patients with missense variants [10,11], mainly because of the very low LDLR activity typical of null variants [12].

Since the cholesterol contained in lipoprotein(a) contributes to LDL-c levels, lipoprotein(a) levels could be an additional factor impacting the phenotypic expression of FH [13]. In fact, a recent study conducted by the Italian network LIPIGEN showed that the correction of LDL-c levels for lipoprotein(a) levels resulted in a lower percentage of FH patients with LDL-c levels ≥190 mg/dL [14].

However, not all patients with clinical suspicion of FH have a pathogenic variant in the canonical genes [6,7], suggesting that elevated levels of LDL-c could have a polygenic cause. Since the first identification of lipid-related loci [15], it was hypothesized that the accumulation of low-impact variants (single nucleotide polymorphisms—SNPs) could lead to increased LDL-c levels similarly to a high-impact variant (monogenic hypercholesterolemia). Several weighted polygenic scores have been developed since then. The first and most studied score was based on 12 SNPs and was developed based on the UK population, with a score range of −0.5–1.46 [16]. Using this score, in patients above the 10th decile of the score distribution (1.16), hypercholesterolemia was considered to have a higher probability to be polygenic. This score was then further refined to reduce the number of SNPs to 6 without losing its performance, according to the original study [17]. Different polygenic scores have been developed to meet the genetic background of different populations, highlighting that a single LDL-c score or a single cut-off cannot be universally used [18,19,20].

Correct genetic identification of the cause of hypercholesterolemia could help patient management, particularly impacting therapy and CVD prevention from childhood onward [1,21]. The aim of our study was to evaluate the impact of two polygenic scores, the 12-SNP score [16] and the 6-SNP score [17], on the predisposition to high LDL-c levels in patients with clinical suspicion of FH, while also considering the presence of FH pathogenic variants.

## 2. Materials and Methods

### 2.1. Patients

A total of 424 patients with a clinical suspicion of FH were recruited at the Dipartimento di Medicina Clinica e Chirurgia and Dipartimento di Scienze Mediche Traslazionali of Università degli Studi di Napoli Federico II and at the Dipartimento di Scienze della Sanità Pubblica e Pediatriche of Università degli Studi di Torino. After the exclusion of six patients with two pathogenic variants, the population consisted of 290 adults (>16 years) and 134 pediatric patients (≤16 years). Most patients were index cases; only 10/290 (3.4%) adults and 7/134 (5.2%) children derived from cascade screening. Most patients were Italian; only 11/424 were from other countries (eight from Europe and three from other continents).

Recruitment criteria are LDL-cholesterol (LDL-c) ≥ 140 mg/dL for adults and LDL-c ≥ 120 mg/dL for children, together with a family history of hypercholesterolemia and/or premature coronary artery disease (<55 years in men or <60 years in women). We included patients with LDL-c levels lower than the usual thresholds of 190 mg/dL for adults and 160 mg/dL for children because we previously observed that some genetically confirmed FH patients may show lower LDL-c levels [11,22].

Written informed consent was collected from each patient. The study was performed according to the current version of the Helsinki Declaration and then approved by the Ethical Committee of the “Università degli Studi di Napoli Federico II” (Number 262/17, 29 November 2017).

### 2.2. Biochemical Data

Total cholesterol, HDL-cholesterol (HDL-c), and triglycerides (TG) were measured using standard enzymatic methods, whereas LDL-c was calculated using the Friedewald formula. Lipid values were measured in the absence of lipid-lowering therapy. If patients were on therapy at the first observation, the pre-therapy LDL-c was estimated using a previously described formula [23]. Non-HDL-cholesterol (non-HDL-c) and the ratios between lipid parameters were also calculated (LDL-c/HDL-c and LDL-c/TG). Since data on lipoprotein(a) were not available for many patients, we did not include this parameter in the analysis.

### 2.3. Genetic Screening

All patients were analyzed by next generation sequencing (NGS) using the Devyser FH v2 kit (Devyser, Sweden), as previously described [11]. Briefly, we enriched the sequence of all exons and the flanking intronic regions of *LDLR*, *APOB*, *PCSK9*, *LDLRAP1*, and *APOE* genes, as well as SNPs for calculating LDL-c polygenic scores. This kit also allowed us to identify copy number variants of the *LDLR* gene. Sequencing was performed using 2 × 150 base pair sequencing with Kit v2 Micro on the MiSeq platform (Illumina, San Diego, CA, USA), and data analysis was performed using Amplicon Suite software version 3.5.1 (SmartSeq). Genes related to FH-phenocopies have not been evaluated.

Variant pathogenicity was evaluated following the American College of Medical Genetics and Genomics (ACMG) guidelines [24], taking into account the recommendation made by Chora et al. in 2018 for variants in *APOB* and *PCSK9* genes [25] and in 2022 for variants in the *LDLR* gene [26]. Rare variants were then divided into uncertain significance variants (USVs) and pathogenic variants. According to the guidelines, USVs are variants with contrasting evidence of pathogenicity or variants for which not enough pathogenicity or benignity criteria are present, implying that new evidence could change the variant classification in the future.

### 2.4. Statistical Analysis

Categorical data were reported as absolute number and percentages, whereas continuous variables were expressed as medians and interquartile ranges (IQRs) because all had non-parametric distribution according to the Kolmogorov–Smirnov test. Frequencies were compared using the exact Fisher test. Continuous variables were compared using the Mann–Whitney or Kruskal–Wallis test in cases of two or more than two groups, respectively. Statistical analyses were performed with IBM SPSS Statistics, Version 29.0 (IBM Corp, Armonk, NY, USA). A *p*-value < 0.05 was considered significant. Receiver operating characteristic (ROC) curves were constructed with MedCalc Statistical Software version 22 (MedCalc Software bv, Ostend, Belgium) using the Hanley and McNeil method to evaluate the significance of the area under the curve (AUC) versus the area under the bisector and to compare different curves. Violin plots were constructed using GraphPad Prism version 10.2 (GraphPad Software, Boston, MA, USA).

## 3. Results

### 3.1. Identification of Monogenic Causes of Hypercholesterolemia

Among 424 patients with a clinical suspicion of FH, we identified 162 patients without pathogenic variants and without USVs (V−/USV−), 36 patients with USVs (V−/USV+), and 226 with pathogenic variants (V+) leading to a mutation detection rate of 53.3%. Six homozygotes/double heterozygotes (HoFH) and 220 heterozygotes were identified. Among the 220 heterozygotes, most carried pathogenic variants in the *LDLR* gene (201; 91.4%), whereas 16 (7.3%) and 3 (1.3%) carried pathogenic variants in *APOB* and *PCSK9* genes, respectively. Among the 6 HoFH, 2 homozygotes and 2 compound heterozygotes for variants in *LDLR* and 2 double heterozygotes (variants in both *LDLR* and *APOB*; variants in both *LDLR* and *PCSK9*) were identified. Since homozygous FH is an extremely severe disease with biochemical and clinical features markedly evident and very different from polygenic hypercholesterolemia, we decided to exclude these patients from subsequent analyses, which were performed on a population of 418 patients.

Demographic and biochemical characteristics of the studied population are reported in Table 1. LDL-c levels were higher in adults versus children, as well as in V+ patients versus V− patients. Notably, in pediatric patients, a higher frequency of patients with FH pathogenic variants was observed compared to adults (Table 1), probably because the hypercholesterolemic phenotype in children is usually not related to secondary factors.

### 3.2. Comparison of LDL-c Polygenic Scores in Patients with Different Genetic Statuses

**Figure 1:**
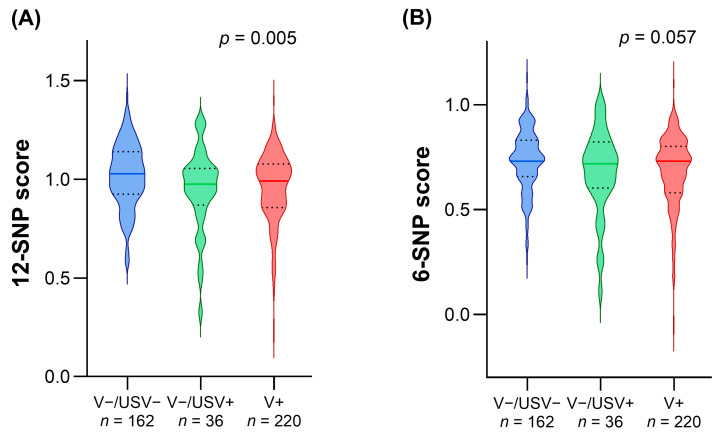
Distribution of the 12-SNP score and 6-SNP score in patients with different genetic statuses. The violin shape represents the smoothed frequency distribution of the 12-SNP score (**A**) and 6-SNP score (**B**) in patients without pathogenic variants and without uncertain significance variants (V−/USV−), without pathogenic variants and with uncertain significance variants (V−/USV+), and with pathogenic variants (V+). The continuous horizontal line within each plot represents the distribution median, whereas the dashed lines represent the first and third quartile of value distribution. Statistical significance indicated in each panel was calculated by Kruskal–Wallis test.

We evaluated the distribution of both the 12-SNP and 6-SNP scores in the three groups defined by genetic status, identifying a significant difference only for the 12-SNP score (Figure 1).

We observed that patients with USVs showed a profile overlapping with both V−/USV− and V+ patients (Figure 1). Since this result can be explained by the main feature of USVs, i.e., the lack of evidence for a definite pathogenicity classification, we excluded this patient group, which represents an undefined genetic status, potentially confusing subsequent analyses.

**Figure 2:**
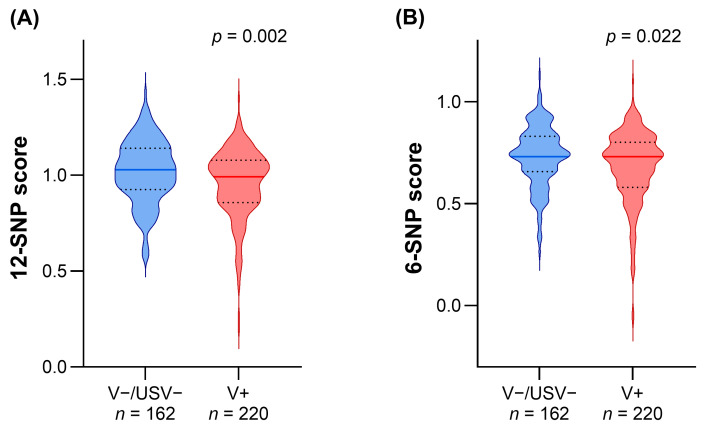
Distribution of the 12-SNP score and 6-SNP score in patients with and without pathogenic variants. The violin shape represents the smoothed frequency distribution of the 12-SNP score (**A**) and 6-SNP score (**B**) in patients without pathogenic variants, without uncertain significance variants (V−/USV−), and with pathogenic variants (V+). The continuous horizontal line within each plot represents the distribution median, whereas the dashed lines represent the first and third quartile of value distribution. Statistical significance indicated in each panel was calculated by Mann–Whitney test.

The comparison of the polygenic scores between V−/USV− and V+ revealed a significant difference for both the 12-SNP and 6-SNP scores (Figure 2). The 12-SNP score was 1.03 (IQR 0.92–1.14) in V−/USV− and 0.99 (IQR 0.86–1.08) in V+ (*p* = 0.002), whereas a lower but still significant difference was observed for the 6-SNP score, which was 0.73 (IQR 0.66–0.83) in V−/USV− and 0.73 (IQR 0.58–0.80) in V+ (*p* = 0.022). Despite a notable overlap of scores, FH/V+ showed a tail in the lowest values of both polygenic scores that was not present in FH/V− patients. However, the 12-SNP score showed more marked differences than the 6-SNP score. We did not perform the analyses dividing adults and pediatric patients because the genetic background is independent of age.

In summary, the results highlighted a different distribution of the 12-SNP and 6-SNP scores between hypercholesterolemic patients with and without FH-causative variants.

### 3.3. Evaluation of a Cut-Off to Identify Patients with a Polygenic Basis of Hypercholesterolemia

**Figure 3:**
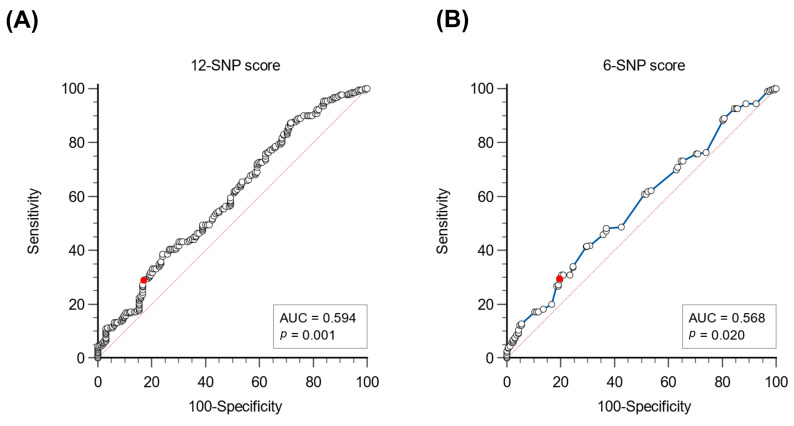
Receiver operating characteristic (ROC) curves of the polygenic scores in relation to the presence of a monogenic cause of FH. (**A**) 12-SNP score; (**B**) 6-SNP score. The curve is illustrated in blue, and each point is depicted as an empty dot, with the exception of the proposed cut-off, which is illustrated in red. The area under the curve (AUC) is reported together with the significance in comparison with the area under the bisector.

An unequivocal cut-off to identify patients with polygenic hypercholesterolemia was not indicated by previous studies. Since all patients included in this study showed hypercholesterolemia and familiarity for hypercholesterolemia, we identified possible cut-offs to distinguish monogenic and polygenic hypercholesterolemia and evaluated their performances. Firstly, we constructed the ROC curve for both 12-SNP and 6-SNP scores, with AUCs of 0.594 (*p* = 0.001) and 0.568 (*p* = 0.020), respectively (Figure 3). The direct comparison revealed a significant difference in AUC (*p* = 0.031), indicating better performance of the 12-SNP score in discriminating monogenic hypercholesterolemia.

Considering that we are facing a population of hypercholesterolemic patients, for each score, we selected a cut-off score value to define 80% of V− as positive for the score, i.e., 0.89 (sensitivity of 31.8% and specificity of 80.2%) for the 12-SNP score and 0.62 (sensitivity of 29.5% and specificity of 80.2%) for the 6-SNP score. Using these cut-offs, the prevalence of patients positive for both the 12-SNP score and the 6-SNP score was lower in V+ patients than in V−/USV− patients (*p* = 0.010 and *p* = 0.033, respectively—Table 2). A larger difference in score positivity prevalence was observed for the 12-SNP score compared with the 6-SNP score.

In summary, a positive score was observed more frequently in patients without pathogenic variants than in patients with FH-causative variants.

### 3.4. Association of Polygenic Scores with Lipid Traits/LDL-Cholesterol Levels

In the whole population, no differences in LDL-c levels were observed when comparing patients with scores above or below the identified cut-offs, i.e., positive or negative for the polygenic scores (Table 3). Considering the groups of patients separately without (V−/USV−) and with pathogenic variants (V+), a significant difference in LDL-c levels between score-positive and score-negative patients was observed only in V+ patients, with a more significant difference for the 12-SNP score compared to the 6-SNP score (Table 3). To also evaluate the role of age and sex on LDL-c levels in relation to score positivity, the analysis was repeated, comparing data by ANCOVA, using age and sex as covariates. For the 12-SNP score, a significant different was now observed in the total population, and it was even more pronounced than before in V+ patients (Table 3).

The linear univariate regression analysis showed that both scores were mainly associated with LDL-c and, to a lesser extent, with total cholesterol and non-HDL cholesterol (Table 4).

Considering that the presence of a pathogenic variant is the major determinant of LDL-c level differences, the same analysis was performed by dividing patients into V−/USV− and V+. The association was still present only in the group of patients with pathogenic variants (V+), with a higher coefficient compared to the total population (Table 4), suggesting that both polygenic scores could be relevant in determining the phenotypic variability observed among genetically determined FH patients.

To further verify this association, we constructed two models of multivariate linear regression using LDL-c levels as the dependent variable and age, sex, the presence of pathogenic variants, and the polygenic score (the 12-SNP score for the first model (Table 5) and the 6-SNP score for the second model (Table 6)) as independent variables. In the total population, the major determinants of LDL-c levels were age and the presence of pathogenic variants, although both scores showed a significant role independently of other factors.

Dividing the population according to the presence of a pathogenic variant, both polygenic scores were associated with LDL-c levels in FH/V+ patients (Table 5 and Table 6), confirming the polygenic influence on the expression of biochemical variability among this group of patients. In the multivariate model, the association of the 12-SNP score with LDL-c levels was also present among FH/V−/USV− patients, although a major role of age was identified.

Polygenic scores were associated with LDL-c levels independently from age, sex, and the presence of pathogenic variants, with substantial differences in adults and children. In fact, when dividing the population into adults and children, the association of both scores with LDL-c levels was more pronounced in children than in adults (Table 5 and Table 6), whereas age was the leading factor influencing LDL-c levels in adults.

Taken together, results confirmed the association of both polygenic scores with LDL-c levels independently of other cholesterol-impacting factors. A greater impact of genetic factors, both monogenic and polygenic, was observed in children.

## 4. Discussion

The prevalence of patients with pathogenic variants among FH-suspected patients is extremely variable, partially depending on the strength of clinical suspicion and the specific population [6,7,27,28,29]. To explain the severe hypercholesterolemia observed in patients without FH-causative variants, the hypothesis of a polygenic basis was formulated [16]. The role of polygenic scores in FH is still debated because great overlap of scores was observed between patients with and without a monogenic cause of FH [16,17,30,31], and no dominant inheritance was observed within families [32].

Since the characterization of the 12-SNP and 6-SNP scores, the 12 SNPs useful for calculating both the 12-SNP and 6-SNP scores have been included in several NGS enrichment panels for FH diagnosis, allowing for the detection of both monogenic and polygenic bases of hypercholesterolemia. Although scores based on several hundreds or thousands of variants could be more tailored for a personalized evaluation of a polygenic trait, especially thanks to the most recent computational methods [33,34,35,36,37], the genetic analysis of these scores would require a cost even higher than the one needed for the screening of monogenic causes of FH, hampering their application in routine diagnosis.

Our study, based on hypercholesterolemic patients with family history of hypercholesterolemia, allowed us to perform analyses aimed at evaluating the ability of polygenic scores to distinguish between monogenic and polygenic hypercholesterolemia. Since FH-causative variants and the SNPs included in the scores are inherited independently of each other, high polygenic scores were present, even in FH/V+ patients, but it is interesting that none of the FH-suspected patients without pathogenic variants showed very low scores. The absence of low polygenic score values in FH-suspected patients without pathogenic variants was also observed in a recent study that analyzed a different score, based on 223 SNPs [31]. The score differences between hypercholesterolemic patients with and without pathogenic variants was the basis for its potential utility and is often observed [16,17].

Several studies were conducted on FH populations after the exclusion of patients with USVs in order to highlight an association with well-defined genetic statuses [30,31,38]. Before doing the same, we also reported the distribution of polygenic scores in V−/USV− patients, highlighting that the score in this group of patients was intermediate between V−/USV− and V+ patients.

As for genetically diagnosed FH patients (FH/V+), our results showed that high values of both the 12-SNP and 6-SNP scores were associated with increased LDL-c levels, representing one of the possible causes of the extreme variability usually observed among FH patients, even among patients sharing the same pathogenic variant [11,39,40,41]. The association of these polygenic scores with LDL-c remains debated according to published studies. In fact, in another Italian study, the association of the 12-SNP score with LDL-c was present in all clinically suspected patients, regardless of the presence of pathogenic variants, although it was more prominent in the presence of pathogenic variants [30]. In a Portuguese study that analyzed a control population to determine the 6-SNP score percentiles, subjects with significantly higher LDL-c levels were observed in the last quartile than in the first quartile, suggesting the use of the 75th percentile (0.76) as a cut-off to define the presence of polygenic hypercholesterolemia [38]. However, no differences in score values were observed between FH patients with and without pathogenic variants [38]. More recently, using the 75th percentile as a cut-off, no differences in LDL-c levels were observed between FH patients with and without pathogenic variants [42]. Also, in a Brazilian population, the association of both 6-SNP and 12-SNP scores with LDL-c levels was observed only in the healthy population and not in the FH group [43].

These discrepancies could be due to the different genetic backgrounds of the different populations, even though the 6-SNP score was tested in seven populations. Our results were obtained from a population composed mainly of Italians. Discordant results among different populations suggest that the polygenic score for LDL-c levels should be tailored to the genetic background of each population. In fact, ethnicity has been demonstrated to be relevant for the distribution of score values and for the association with LDL-c levels [20,43].

We then considered both the presence of pathogenic variants and polygenic scores together with demographic features impacting cholesterol levels to verify the association between polygenic scores and LDL-c levels. In children, the presence of a pathogenic variant was the most relevant factor influencing LDL-c levels, with a considerable role played by both polygenic scores, suggesting that genetic predisposition based on monogenic and polygenic data should be greatly considered in the evaluation of cumulative LDL-c from childhood. On the other hand, in adults, age emerged as the factor with the greatest impact, although the association of the presence of pathogenic variants and both polygenic scores were still present. We previously observed similar differences between adults and children [11], probably due to a greater impact of lifestyle modifications, especially diet-related ones, occurring with age.

Children can be considered the best population group for estimating the role of genetic determinants of hypercholesterolemia, both monogenic and polygenic, because their LDL-c levels are less influenced by the physiological cholesterol increase observed with the advancement of age and by unhealthy lifestyle factors, which are less frequent in children than in adults. In fact, when comparing adults and children, the impact of both the 12-SNP and 6-SNP scores on LDL-c levels was greater in children than in adults. We did not observe any impact of sex on LDL-c levels, although it is well-known that male sex is associated with increased cardiovascular risk; this aspect sometime induces undertreatment of women [44].

As for other lipid traits, we observed that patients without pathogenic variants showed slightly higher triglyceride levels than patients with pathogenic variants, suggesting a potential combined hyperlipidemia as the actual dyslipidemia in this group. We and other groups have previously observed that among patients with a clinical suspicion of familial combined hyperlipidemia (FCH), several patients with FH-causative variants were identified [45,46], highlighting the biochemical and clinical overlap of these two diseases. FCH is a polygenic disease in which different SNPs have been implicated for their association with LDL-c and triglyceride levels [47,48]. 

Only a modest association of both scores was observed with the LDL/HDL ratio, which in children emerged as the best parameter to discriminate patients with and without pathogenic variants [22].

Our study also allows for a direct comparison of the performance of the 12-SNP and 6 SNP scores. Firstly, when comparing patients with and without pathogenic variants, a greater difference was observed in the 12-SNP score compared to the 6-SNP score. This result could be explained by the wider number of score combinations that can be obtained with a larger number of SNPs, allowing for a more detailed definition of the score.

The better performance of the 12-SNP score compared to the 6-SNP score was also proven by the analysis of patients positive and negative, considering cut-off score values associated with a detection of 80% of score-positive patients in V−/USV− (0.89 for the 12-SNP score and 0.62 for the 6-SNP score). Using these cut-offs, a positive score was observed more frequently in V−/USV− patients than in V+ patients, indicating that both polygenic scores could be considered as an alternative cause of hypercholesterolemia when no monogenic causes are present. However, despite the same specificity, the sensitivity of the 12-SNP score was higher, as was the significance level. This result, together with the direct comparison of ROC curves, further confirms that the 12-SNP score showed greater power in discriminating polygenic from monogenic hypercholesterolemic patients.

Most of the studies on the 12-SNP and 6-SNP scores have defined their own cut-offs based on the studied population [20,30,31,38]. Defining a single cut-off valid for all populations is even more difficult than using the same polygenic score in different populations.

The clinical utility of polygenic scores was also evaluated with respect to predisposition to CVD, allowing for accurate risk stratification. It has been well-demonstrated that the presence of a monogenic cause of FH is associated with an increased risk for CVD events, independently of LDL-c levels, as well as for subclinical atherosclerosis [10,29,49]. Among patients with a monogenic cause of hypercholesterolemia, positivity to a score based on 28 SNPs was associated with increased CVD risk, whereas this association was not present in the absence of FH-causative variants [50]. Using another score based on 223 SNPs, a progressive increase in CVD risk was observed from genetically negative patients to patients with polygenic hypercholesterolemia and to patients with monogenic hypercholesterolemia [51].

Polygenic risk scores could also be integrated with functional evaluations, including LDL uptake and lipid mobilization in peripheral blood cells, to construct hybrid scores useful for identifying subjects more prone to hypercholesterolemia [52]. Also, integration of polygenic scores with biochemical data, in particular, lipoprotein(a) levels that are genetically determined, could be used to evaluate the role of all possible genetic causes of hypercholesterolemia [31,42]. Extended genetic screening, including the evaluation of other genes associated with hypercholesterolemia, could further enhance the identification of patients at high risk of hypercholesterolemia.

Several innovative cholesterol-lowering therapies have been developed for FH patients, mainly based on PCSK9 inhibition through antibodies or small interfering RNA [53,54,55]. Future studies could focus on cost–benefit analysis about the use of these therapies in patients with polygenic hypercholesterolemia.

Study limitations include the lack of a control population to establish percentiles of 12-SNP and 6-SNP scores in the Italian population and the lack of genetic screening for genes causative of other dyslipidemias that could be FH-phenocopies. In addition, due to the lack of data, the impact of high lipoprotein(a) levels on FH clinical suspicion was not evaluated.

In conclusion, high polygenic scores should be considered as predisposing factors to hypercholesterolemia, with a lower impact on the phenotype than an FH-causative variant but still notable. Furthermore, polygenic scores can also modulate the phenotype in patients with a monogenic cause of FH, integrating the definition of the genetic background. Research efforts are still required to translate polygenic scores into clinical practice, weighting the impact of scores in determining high LDL-c levels both in the general population and in genetically diagnosed FH patients.

## Figures and Tables

**Table 1:** Demographic and biochemical characteristics of studied patients with comparisons between adults and children and between patients with (FH/V+) and without (FH/V−) pathogenic variants.

	Total FH Patients *n* = 418	Adults *n* = 285	Children *n* = 133	Significance ^1^	FH/V+ Patients *n* = 220	FH/V− Patients *n* = 198	Significance ^2^
Age (years)	31 (14–54)	47 (30–58)	11 (9–13)	*p* < 0.001	20 (12–44)	45 (21–58)	*p* < 0.001
Sex *n* males (%)	188.0 (45%)	120.0 (42.1%)	68.0 (51.1%)	ns	103.0 (46.8%)	85.0 (42.9%)	ns
Pediatric patients *n* (%)	133.0 (31.8%)	-	-	-	91.0 (41.4%)	42.0 (21.2%)	*p* < 0.001
LDL-cholesterol (mg/dL)	209 (176–251)	213 (181–260)	198 (155–232)	*p* < 0.001	215 (183–263)	198 (172–234)	*p* = 0.004
Total cholesterol (mg/dL)	295 (253–345)	303 (262–367)	271 (230–312)	*p* < 0.001	299 (253–350)	293 (253–332)	ns
HDL-cholesterol (mg/dL)	52 (44–62)	51 (43–62)	54 (47–64)	*p* = 0.041	52 (45–61)	53 (44–67)	ns
Non HDL-cholesterol (mg/dL)	241 (200–292)	249 (211–313)	214 (177–257)	*p* < 0.001	245 (203–298)	236 (198–284)	ns
Triglycerides (mg/dL)	102 (75–155)	114 (84–166)	82 (65–110)	*p* < 0.001	91 (67–142)	114 (86–166)	*p* < 0.001
LDL/HDL ratio	4.0 (3.1–5.2)	4.3 (3.4–5.5)	3.5 (2.8–4.5)	*p* < 0.001	4.2 (3.2–5.4)	3.9 (2.9–4.9)	*p* = 0.012
Presence of pathogenic variants *n* (%)	220.0 (52.6%)	129.0 (45.3%)	91.0 (68.4%)	*p* < 0.001	-	-	-

^1^ Comparison of adults and children. ^2^ Comparison of FH/V+ and FH/V−. ns = not significant.

**Table 2:** Frequency of patients positive for the 12-SNP and 6-SNP scores in groups defined by the presence (FH/V+) or absence of pathogenic variants/USV (FH/V−/USV−).

	FH/V−/USV− *n* = 162	FH/V+ *n* = 220	Significance
12-SNP score −	32 (19.8%)	70 (31.2%)	*p* = 0.010
12-SNP score +	130 (80.2%)	150 (68.2%)	
6-SNP score −	32 (19.8%)	65 (29.5%)	*p* = 0.033
6-SNP score +	130 (80.2%)	155 (70.5%)	

**Table 3:** Comparison of LDL-c levels between patients positive and negative for 12-SNP and 6-SNP scores.

	Total FH Patients *n* = 382	Mann-Whitney ^1^	ANCOVA ^2^	FH/V−/USV− *n* = 162	Mann-Whitney ^1^	ANCOVA ^2^	FH/V+ *n* = 220	Mann-Whitney ^1^	ANCOVA ^2^
**12-SNP score −**	210 (165–239)	ns	*p* = 0.027	204 (169–231)	ns	ns	212 (162–240)	*p* = 0.017	*p* = 0.006
**12-SNP score +**	209 (177–261)			197 (173–236)			223 (187–279)		
**6-SNP score −**	213 (163–241)	ns	ns	204 (166–231)	ns	ns	215 (159–246)	*p* = 0.044	*p* = 0.048
**6-SNP score +**	209 (178–259)			197 (173–236)			217 (187–279)		

^1^ Significance at Mann-Whitney comparing score − vs. score +. ^2^ Significance at ANCOVA comparing score − vs. score + using age and sex as covariates. ns = not significant.

**Table 4:** Association of polygenic scores with lipid parameters by univariate linear regression.

	Total FH Patients *n* = 382				FH/V−/USV− *n* = 162				FH/V+ *n* = 220			
	12-SNP Score		6-SNP Score		12-SNP Score		6-SNP Score		12-SNP Score		6-SNP Score	
	β-Coefficient	*p*-Value	β-Coefficient	*p*-Value	β-Coefficient	*p*-Value	β-Coefficient	*p*-Value	β-Coefficient	*p*-Value	β-Coefficient	*p*-Value
LDL-cholesterol (mg/dL)	0.144	*p* = 0.005	0.134	*p* = 0.009	0.104	ns	0.032	ns	0.212	*p* = 0.002	0.227	*p* < 0.001
Total cholesterol (mg/dL)	0.120	*p* = 0.020	0.111	*p* = 0.031	0.072	ns	0.021	ns	0.169	*p* = 0.012	0.183	*p* = 0.007
HDL-cholesterol (mg/dL)	−0.003	ns	−0.003	ns	0.055	ns	0.092	ns	−0.069	ns	−0.089	ns
Non-HDL cholesterol (mg/dL)	0.127	*p* = 0.014	0.118	*p* = 0.022	0.068	ns	0.008	ns	0.186	*p* = 0.006	0.203	*p* = 0.002
Triglycerides (mg/dL)	0.045	ns	0.026	ns	−0.015	ns	−0.050	ns	0.026	ns	0.030	ns
LDL/HDL ratio	0.089	ns	0.085	ns	0.041	ns	−0.029	ns	0.157	*p* = 0.021	0.186	*p* = 0.006

ns = not significant.

**Table 5:** Factors associated with LDL-c levels by multivariate linear regression, including the 12-SNP score in the total population and in groups defined by the presence (V+) or absence of pathogenic variants (V−/USV−) or in adult/pediatric patients.

	Total Population *n* = 382		FH/V+ Patients *n* = 220		FH/V−/USV− Patients *n* = 160		Adults *n* = 257		Children *n* = 125	
Independent Variables	β	Significance	β	Significance	β	Significance	β	Significance	β	Significance
Age	0.274	*p* < 0.001	0.151	*p* = 0.025	0.415	*p* < 0.001	0.218	*p* = 0.001	−0.052	ns
Sex	0.017	ns	0.044	ns	−0.010	ns	0.023	ns	0.043	ns
Presence of pathogenic variants	0.240	*p* < 0.001	-	-	-	-	0.175	*p* = 0.007	0.407	*p* < 0.001
12-SNP score	0.169	*p* = 0.001	0.199	*p* = 0.003	0.154	*p* = 0.037	0.149	*p* = 0.016	0.242	*p* = 0.005

ns = not significant.

**Table 6:** Factors associated with LDL-c levels by multivariate linear regression, including the 6-SNP score in total population and in groups defined by the presence (V+) or absence of pathogenic variants (V−/USV−) or in adult/pediatric patients.

	Total Population *n* = 382		FH/V+ Patients *n* = 220		FH/V−/USV− Patients *n* = 160		Adults *n* = 257		Children *n* = 125	
Independent Variables	β	Significance	β	Significance	β	Significance	β	Significance	β	Significance
Age	0.273	*p* < 0.001	0.149	*p* = 0.027	0.405	*p* < 0.001	0.213	*p* = 0.001	−0.043	ns
Sex	0.017	ns	0.038	ns	0.003	ns	0.027	ns	0.029	ns
Presence of pathogenic variants	0.233	*p* < 0.001	-	-	-	-	0.166	*p* = 0.009	0.400	*p* < 0.001
6-SNP score	0.152	*p* = 0.002	0.211	*p* = 0.002	0.083	ns	0.146	*p* = 0.017	0.201	*p* = 0.021

ns = not significant.

## Data Availability

Data are available from the corresponding author upon reasonable request.

## References

[1] Watts G.F., Gidding S.S., Hegele R.A., Raal F.J., Sturm A.C., Jones L.K., Sarkies M.N., Al-Rasadi K., Blom D.J., Daccord M. (2023). International Atherosclerosis Society Guidance for Implementing Best Practice in the Care of Familial Hypercholesterolaemia. Nat. Rev. Cardiol..

[2] Akioyamen L.E., Genest J., Shan S.D., Reel R.L., Albaum J.M., Chu A., Tu J.V. (2017). Estimating the Prevalence of Heterozygous Familial Hypercholesterolaemia: A Systematic Review and Meta-Analysis. BMJ Open.

[3] Tromp T.R., Hartgers M.L., Hovingh G.K., Vallejo-Vaz A.J., Ray K.K., Soran H., Freiberger T., Bertolini S., Harada-Shiba M., Blom D.J. (2022). Worldwide Experience of Homozygous Familial Hypercholesterolaemia: Retrospective Cohort Study. Lancet.

[4] Di Taranto M.D., Giacobbe C., Buonaiuto A., Calcaterra I., Palma D., Maione G., Iannuzzo G., Di Minno M.N.D., Rubba P., Fortunato G. (2020). A Real-World Experience of Clinical, Biochemical and Genetic Assessment of Patients with Homozygous Familial Hypercholesterolemia. J. Clin. Med..

[5] Alves A.C., Alonso R., Diaz-Diaz J.L., Medeiros A.M., Jannes C.E., Merchan A., Vasques-Cardenas N.A., Cuevas A., Chacra A.P., Krieger J.E. (2020). Phenotypical, Clinical, and Molecular Aspects of Adults and Children With Homozygous Familial Hypercholesterolemia in Iberoamerica. Arterioscler. Thromb. Vasc. Biol..

[6] Di Taranto M.D., Fortunato G. (2023). Genetic Heterogeneity of Familial Hypercholesterolemia: Repercussions for Molecular Diagnosis. Int. J. Mol. Sci..

[7] Abifadel M., Boileau C. (2023). Genetic and Molecular Architecture of Familial Hypercholesterolemia. J. Intern. Med..

[8] Tada H., Okada H., Nomura A., Yashiro S., Nohara A., Ishigaki Y., Takamura M., Kawashiri M.-A. (2019). Rare and Deleterious Mutations in ABCG5/ABCG8 Genes Contribute to Mimicking and Worsening of Familial Hypercholesterolemia Phenotype. Circ. J..

[9] Tada H., Nohara A., Kawashiri M.-A. (2019). Monogenic, Polygenic, and Oligogenic Familial Hypercholesterolemia. Curr. Opin. Lipidol..

[10] Khera A.V., Won H.-H., Peloso G.M., Lawson K.S., Bartz T.M., Deng X., van Leeuwen E.M., Natarajan P., Emdin C.A., Bick A.G. (2016). Diagnostic Yield and Clinical Utility of Sequencing Familial Hypercholesterolemia Genes in Patients With Severe Hypercholesterolemia. J. Am. Coll. Cardiol..

[11] Di Taranto M.D., Giacobbe C., Palma D., Iannuzzo G., Gentile M., Calcaterra I., Guardamagna O., Auricchio R., Di Minno M.N.D., Fortunato G. (2021). Genetic Spectrum of Familial Hypercholesterolemia and Correlations with Clinical Expression: Implications for Diagnosis Improvement. Clin. Genet..

[12] Romano M., Di Taranto M.D., Mirabelli P., D’Agostino M.N., Iannuzzi A., Marotta G., Gentile M., Raia M., Di Noto R., Del Vecchio L. (2011). An Improved Method on Stimulated T-Lymphocytes to Functionally Characterize Novel and Known LDLR Mutations. J. Lipid Res..

[13] Kronenberg F., Mora S., Stroes E.S.G., Ference B.A., Arsenault B.J., Berglund L., Dweck M.R., Koschinsky M., Lambert G., Mach F. (2022). Lipoprotein(a) in Atherosclerotic Cardiovascular Disease and Aortic Stenosis: A European Atherosclerosis Society Consensus Statement. Eur. Heart J..

[14] Olmastroni E., Gazzotti M., Averna M., Arca M., Tarugi P., Calandra S., Bertolini S., Catapano A.L., Casula M., LIPIGEN Study Group (2023). Lipoprotein(a) Genotype Influences the Clinical Diagnosis of Familial Hypercholesterolemia. J. Am. Heart Assoc..

[15] Teslovich T.M., Musunuru K., Smith A.V., Edmondson A.C., Stylianou I.M., Koseki M., Pirruccello J.P., Ripatti S., Chasman D.I., Willer C.J. (2010). Biological, Clinical and Population Relevance of 95 Loci for Blood Lipids. Nature.

[16] Talmud P.J., Shah S., Whittall R., Futema M., Howard P., Cooper J.A., Harrison S.C., Li K., Drenos F., Karpe F. (2013). Use of Low-Density Lipoprotein Cholesterol Gene Score to Distinguish Patients with Polygenic and Monogenic Familial Hypercholesterolaemia: A Case-Control Study. Lancet.

[17] Futema M., Shah S., Cooper J.A., Li K., Whittall R.A., Sharifi M., Goldberg O., Drogari E., Mollaki V., Wiegman A. (2015). Refinement of Variant Selection for the LDL Cholesterol Genetic Risk Score in the Diagnosis of the Polygenic Form of Clinical Familial Hypercholesterolemia and Replication in Samples from 6 Countries. Clin. Chem..

[18] Wang J., Dron J.S., Ban M.R., Robinson J.F., McIntyre A.D., Alazzam M., Zhao P.J., Dilliott A.A., Cao H., Huff M.W. (2016). Polygenic Versus Monogenic Causes of Hypercholesterolemia Ascertained Clinically. Arterioscler. Thromb. Vasc. Biol..

[19] Tada H., Yeo K.K., Li J.-J., Tan K., Ako J., Krittayaphong R., San Tan R., Aylward P.E., Lam C.S.P., Baek S.H. (2021). Polygenic Risk Scores for Atherosclerotic Cardiovascular Disease in the Asia-Pacific Region. JACC Asia.

[20] Gratton J., Finan C., Hingorani A.D., Humphries S.E., Futema M. (2022). LDL-C Concentrations and the 12-SNP LDL-C Score for Polygenic Hypercholesterolaemia in Self-Reported South Asian, Black and Caribbean Participants of the UK Biobank. Front. Genet..

[21] Wiegman A., Gidding S.S., Watts G.F., Chapman M.J., Ginsberg H.N., Cuchel M., Ose L., Averna M., Boileau C., Borén J. (2015). Familial Hypercholesterolaemia in Children and Adolescents: Gaining Decades of Life by Optimizing Detection and Treatment. Eur. Heart J..

[22] Di Taranto M.D., de Falco R., Guardamagna O., Massini G., Giacobbe C., Auricchio R., Malamisura B., Proto M., Palma D., Greco L. (2019). Lipid Profile and Genetic Status in a Familial Hypercholesterolemia Pediatric Population: Exploring the LDL/HDL Ratio. Clin. Chem. Lab. Med..

[23] Langsted A., Kamstrup P.R., Benn M., Tybjærg-Hansen A., Nordestgaard B.G. (2016). High Lipoprotein(a) as a Possible Cause of Clinical Familial Hypercholesterolaemia: A Prospective Cohort Study. Lancet Diabetes Endocrinol..

[24] Richards S., Aziz N., Bale S., Bick D., Das S., Gastier-Foster J., Grody W.W., Hegde M., Lyon E., Spector E. (2015). Standards and Guidelines for the Interpretation of Sequence Variants: A Joint Consensus Recommendation of the American College of Medical Genetics and Genomics and the Association for Molecular Pathology. Genet. Med..

[25] Chora J.R., Medeiros A.M., Alves A.C., Bourbon M. (2018). Analysis of Publicly Available LDLR, APOB, and PCSK9 Variants Associated with Familial Hypercholesterolemia: Application of ACMG Guidelines and Implications for Familial Hypercholesterolemia Diagnosis. Genet. Med..

[26] Chora J.R., Iacocca M.A., Tichý L., Wand H., Kurtz C.L., Zimmermann H., Leon A., Williams M., Humphries S.E., Hooper A.J. (2022). The Clinical Genome Resource (ClinGen) Familial Hypercholesterolemia Variant Curation Expert Panel Consensus Guidelines for LDLR Variant Classification. Genet. Med..

[27] Rubba P., Gentile M., Marotta G., Iannuzzi A., Sodano M., De Simone B., Jossa F., Iannuzzo G., Giacobbe C., Di Taranto M.D. (2017). Causative Mutations and Premature Cardiovascular Disease in Patients with Heterozygous Familial Hypercholesterolaemia. Eur. J. Prev. Cardiol..

[28] Saadatagah S., Alhalabi L., Farwati M., Zordok M., Bhat A., Smith C.Y., Wood-Wentz C.M., Bailey K.R., Kullo I.J. (2022). The Burden of Severe Hypercholesterolemia and Familial Hypercholesterolemia in a Population-Based Setting in the US. Am. J. Prev. Cardiol..

[29] D’Erasmo L., Minicocci I., Di Costanzo A., Pigna G., Commodari D., Ceci F., Montali A., Brancato F., Stanca I., Nicolucci A. (2021). Clinical Implications of Monogenic Versus Polygenic Hypercholesterolemia: Long-Term Response to Treatment, Coronary Atherosclerosis Burden, and Cardiovascular Events. J. Am. Heart Assoc..

[30] Olmastroni E., Gazzotti M., Arca M., Averna M., Pirillo A., Catapano A.L., Casula M., LIPIGEN Study Group (2022). Twelve Variants Polygenic Score for Low-Density Lipoprotein Cholesterol Distribution in a Large Cohort of Patients With Clinically Diagnosed Familial Hypercholesterolemia With or Without Causative Mutations. J. Am. Heart Assoc..

[31] Schwaninger G., Forer L., Ebenbichler C., Dieplinger H., Kronenberg F., Zschocke J., Witsch-Baumgartner M. (2023). Filling the Gap: Genetic Risk Assessment in Hypercholesterolemia Using LDL-C and LPA Genetic Scores. Clin. Genet..

[32] Sjouke B., Tanck M.W.T., Fouchier S.W., Defesche J.C., Hutten B.A., Wiegman A., Kastelein J.J.P., Hovingh G.K. (2016). Children with Hypercholesterolemia of Unknown Cause: Value of Genetic Risk Scores. J. Clin. Lipidol..

[33] Ripatti P., Rämö J.T., Mars N.J., Fu Y., Lin J., Söderlund S., Benner C., Surakka I., Kiiskinen T., Havulinna A.S. (2020). Polygenic Hyperlipidemias and Coronary Artery Disease Risk. Circ. Genom. Precis. Med..

[34] Sinnott-Armstrong N., Tanigawa Y., Amar D., Mars N., Benner C., Aguirre M., Venkataraman G.R., Wainberg M., Ollila H.M., Kiiskinen T. (2021). Genetics of 35 Blood and Urine Biomarkers in the UK Biobank. Nat. Genet..

[35] Graham S.E., Clarke S.L., Wu K.-H.H., Kanoni S., Zajac G.J.M., Ramdas S., Surakka I., Ntalla I., Vedantam S., Winkler T.W. (2021). The Power of Genetic Diversity in Genome-Wide Association Studies of Lipids. Nature.

[36] Camastra F., Di Taranto M.D., Staiano A. (2015). Statistical and Computational Methods for Genetic Diseases: An Overview. Comput. Math. Methods Med..

[37] Zhang H., Zhan J., Jin J., Zhang J., Lu W., Zhao R., Ahearn T.U., Yu Z., O’Connell J., Jiang Y. (2023). A New Method for Multiancestry Polygenic Prediction Improves Performance across Diverse Populations. Nat. Genet..

[38] Mariano C., Alves A.C., Medeiros A.M., Chora J.R., Antunes M., Futema M., Humphries S.E., Bourbon M. (2020). The Familial Hypercholesterolaemia Phenotype: Monogenic Familial Hypercholesterolaemia, Polygenic Hypercholesterolaemia and Other Causes. Clin. Genet..

[39] Cuchel M., Bruckert E., Ginsberg H.N., Raal F.J., Santos R.D., Hegele R.A., Kuivenhoven J.A., Nordestgaard B.G., Descamps O.S., Steinhagen-Thiessen E. (2014). Homozygous Familial Hypercholesterolaemia: New Insights and Guidance for Clinicians to Improve Detection and Clinical Management. A Position Paper from the Consensus Panel on Familial Hypercholesterolaemia of the European Atherosclerosis Society. Eur. Heart J..

[40] Gazzotti M., Casula M., Bertolini S., Capra M.E., Olmastroni E., Catapano A.L., Pederiva C., LIPIGEN Paediatric Group (2022). The Role of Registers in Increasing Knowledge and Improving Management of Children and Adolescents Affected by Familial Hypercholesterolemia: The LIPIGEN Pediatric Group. Front. Genet..

[41] Awan Z.A., Rashidi O.M., Al-Shehri B.A., Jamil K., Elango R., Al-Aama J.Y., Hegele R.A., Banaganapalli B., Shaik N.A. (2021). Saudi Familial Hypercholesterolemia Patients With Rare LDLR Stop Gain Variant Showed Variable Clinical Phenotype and Resistance to Multiple Drug Regimen. Front. Med..

[42] Medeiros A.M., Alves A.C., Miranda B., Chora J.R., Bourbon M., Investigators of the Portuguese FH Study (2024). Unraveling the Genetic Background of Individuals with a Clinical Familial Hypercholesterolemia Phenotype. J. Lipid Res..

[43] Lima I.R., Tada M.T., Oliveira T.G.M., Jannes C.E., Bensenor I., Lotufo P.A., Santos R.D., Krieger J.E., Pereira A.C. (2022). Polygenic Risk Score for Hypercholesterolemia in a Brazilian Familial Hypercholesterolemia Cohort. Atheroscler. Plus.

[44] Zamora A., Ramos R., Comas-Cufi M., García-Gil M., Martí-Lluch R., Plana N., Alves-Cabratosa L., Ponjoan A., Rodríguez-Borjabad C., Ibarretxe D. (2023). Women with Familial Hypercholesterolemia Phenotype Are Undertreated and Poorly Controlled Compared to Men. Sci. Rep..

[45] Staiano A., di Taranto M.D., Bloise E., D’Agostino M.N., D’Angelo A., Marotta G., Gentile M., Jossa F., Iannuzzi A., Rubba P. (2013). Investigation of Single Nucleotide Polymorphisms Associated to Familial Combined Hyperlipidemia with Random Forests. Neural Nets and Surroundings.

[46] Civeira F., Jarauta E., Cenarro A., García-Otín A.L., Tejedor D., Zambón D., Mallen M., Ros E., Pocoví M. (2008). Frequency of Low-Density Lipoprotein Receptor Gene Mutations in Patients with a Clinical Diagnosis of Familial Combined Hyperlipidemia in a Clinical Setting. J. Am. Coll. Cardiol..

[47] Dron J.S. (2023). The Clinical Utility of Polygenic Risk Scores for Combined Hyperlipidemia. Curr. Opin. Lipidol..

[48] Ripatti P., Rämö J.T., Söderlund S., Surakka I., Matikainen N., Pirinen M., Pajukanta P., Sarin A.-P., Service S.K., Laurila P.-P. (2016). The Contribution of GWAS Loci in Familial Dyslipidemias. PLoS Genet..

[49] Di Minno M.N.D., Gentile M., Di Minno A., Iannuzzo G., Calcaterra I., Buonaiuto A., Di Taranto M.D., Giacobbe C., Fortunato G., Rubba P.O.F. (2020). Changes in Carotid Stiffness in Patients with Familial Hypercholesterolemia Treated with Evolocumab^®^: A Prospective Cohort Study. Nutr. Metab. Cardiovasc. Dis..

[50] Trinder M., Li X., DeCastro M.L., Cermakova L., Sadananda S., Jackson L.M., Azizi H., Mancini G.B.J., Francis G.A., Frohlich J. (2019). Risk of Premature Atherosclerotic Disease in Patients With Monogenic Versus Polygenic Familial Hypercholesterolemia. J. Am. Coll. Cardiol..

[51] Trinder M., Francis G.A., Brunham L.R. (2020). Association of Monogenic vs Polygenic Hypercholesterolemia With Risk of Atherosclerotic Cardiovascular Disease. JAMA Cardiol..

[52] Pfisterer S.G., Brock I., Kanerva K., Hlushchenko I., Paavolainen L., Ripatti P., Islam M.M., Kyttälä A., Di Taranto M.D., Scotto di Frega A. (2022). Multiparametric Platform for Profiling Lipid Trafficking in Human Leukocytes. Cell Rep. Methods.

[53] Kastelein J.J.P., Ginsberg H.N., Langslet G., Hovingh G.K., Ceska R., Dufour R., Blom D., Civeira F., Krempf M., Lorenzato C. (2015). ODYSSEY FH I and FH II: 78 Week Results with Alirocumab Treatment in 735 Patients with Heterozygous Familial Hypercholesterolaemia. Eur. Heart J..

[54] Giugliano R.P., Pedersen T.R., Park J.-G., De Ferrari G.M., Gaciong Z.A., Ceska R., Toth K., Gouni-Berthold I., Lopez-Miranda J., Schiele F. (2017). Clinical Efficacy and Safety of Achieving Very Low LDL-Cholesterol Concentrations with the PCSK9 Inhibitor Evolocumab: A Prespecified Secondary Analysis of the FOURIER Trial. Lancet.

[55] Raal F.J., Kallend D., Ray K.K., Turner T., Koenig W., Wright R.S., Wijngaard P.L.J., Curcio D., Jaros M.J., Leiter L.A. (2020). Inclisiran for the Treatment of Heterozygous Familial Hypercholesterolemia. N. Engl. J. Med..

